# Analyzing assisted reproductive treatment representations in Italy and Spain through newspapers

**DOI:** 10.3389/fpsyg.2024.1451663

**Published:** 2024-10-23

**Authors:** Marta Anna Stella Vizzini, Silvia Monaco, Loredana Tetecher, Claudia Cappadonna, Vincenzo Ambriola, Michela Di Trani, Rachele Mariani

**Affiliations:** Department of Dynamic and Clinical Psychology and Health Studies, “Sapienza” University of Rome, Rome, Italy

**Keywords:** assisted reproductive treatments, infertility, representations, newspapers, cultures, Italy, Spain

## Abstract

With the increase in infertility cases recorded over the last 20 years, there is a considerable demand for assisted reproductive treatments (ART). However, there is significant variation in the availability of such treatments across different countries. Legislation on assisted reproduction is influenced by various cultural expressions, making it seemingly impossible to create a single representation adaptable to different contexts. This work investigates the cultural representations of ART in Italy and Spain. We collected 1,735 articles from two Italian and Spanish newspapers, with all the articles containing the respective translation of ART from 2013 to 2022. The two corpora were analyzed using the Emotional Text Mining (ETM) methodology. The analysis produced 3 clusters in the Italian corpus and 5 clusters in the Spanish corpus. From the Italian results, a view of ART emerged that is linked to ethical limitations and the ideal of the traditional family. In contrast, the Spanish results depict ART in terms of community, rights, public health, and birth seen in itself. In conclusion, this study highlights the strong differences between Italian and Spanish cultures regarding ART. The results could be used to improve clinical practices and legislation surrounding ART.

## 1 Introduction

There is an unstoppable increase in demand for Assisted Reproductive Treatment (ART) (Gianaroli et al., 2016) due to the rise in infertility cases over the last two decades (Ministero della Salute, 2022). The average global infertility rate is 10% (Direkvand Moghaddam et al., 2016), while in Italy, the prevalence of infertility is estimated to be around 15–20% (Istituto Superiore di Sanità, 2020). Additionally, a crucial factor is the increase in the average age of women at first childbirth over the last four decades by 3.7 years, a phenomenon closely linked to the growing tendency to postpone parenthood (Mills et al., 2021). The number of children conceived by ART currently exceeds 4 million, accounting for 0.1% of the world’s population (Faddy et al., 2018).

Assisted reproductive treatments legislation is influenced by a variety of factors, including social structure, political choices, ethical issues, and religious beliefs. Consequently, it often triggers ethical and moral discussions related to the body and societal norms, making it challenging to establish a standard regulation adaptable across different countries (Gianaroli et al., 2016).

A previous analysis of Italian culture regarding infertility and ART (Cordella et al., 2018) highligthed that infertility is often pathologized, with conception shifting from a private to a medicalized event. A comparative study found that Italy has the lowest level of support for gamete donation among European countries, largely influenced by Catholic doctrine (Fauser et al., 2019). In contrast, Spain, despite its Catholic presence, has adopted more liberal ART policies (Alon and Pinilla, 2021). This shift is attributed to Spain’s historical approach to reproductive health, viewed as both a health and moral issue (Bravo-Moreno, 2017). Currently, Spain leads Europe in ART, particularly in fertility treatments (Smeenk et al., 2023), with strong support for IVF for single women, same-sex couples, and gamete donation, unlike Italy, which remains the least supportive on these issues (Fauser et al., 2019). In Spain, the economic aspect of ART also plays a significant role. A Spanish article reported that only individuals with sufficient financial resources can access the more effective ART techniques (Alon and Pinilla, 2021). Meanwhile, the narrative promoted by private clinics suggests that ART enables individuals to ‘delay’ childbearing through IVF (Mohammadi et al., 2019).

These differences in attitudes toward ART can be explained by the different historical and political paths that led to the legislation in the two countries. In Italy, Law 40/2004 emphasizes “embryo protection,” reflecting a bioethical stance rooted in Catholic culture (Nappi et al., 2000; Robertson, 2004). In contrast, Spain’s response to the severe economic and demographic crisis of 2008 led to greater openness in its healthcare system toward ART as a means of fostering economic development (Alon et al., 2019).

Assisted reproductive treatments regularly attracts media attention, which significantly influences collective perceptions and social representations (De La Rochebrochard, 2021). The press plays a well-documented role in shaping representations and conveying meanings (Cordella, 2016; Giacchetta et al., 2016; Greco, 2016a,b). Numerous studies have examined newspaper coverage to understand the social portrayal of various topics, including ART (Raynor et al., 2017; Rizzoli et al., 2017; Young et al., 2017).

In conclusion, the expression of both demand and supply is a function of cultural context (Storr, 2010). Studying social representation is one way to understand cultural factors that may influence opinions (Greco and Polli, 2020). Articles published in newspapers are considered indicators of socially shared representation on a topic (Carli, 1990) as they must use the same symbolic-cultural system to communicate with their readers (Greco, 2016a,b). For this reason, we can consider them a proxy for the cultural context present in the country (Greco, 2016b; Cordella et al., 2018; Greco and Polli, 2020).

Despite the significant role of culture in shaping emotions, attitudes, and behaviors toward ART, no study has yet explored the underlying factors contributing to the differences in legislation, beliefs, and practices between the two countries.

The present study aims to explore the emerging representations in Italian and Spanish cultures as reflected in newspaper writings, highlighting the main socio-cultural differences. We hypothesize that both cultural and legislative dimensions can be traced in the narratives reported by newspapers, highlighting the link between religious and cultural aspects and access to ART in both countries. By comparing the emerging cultures of the two countries, it will be possible to shed light on how these aspects may be profoundly linked.

## 2 Materials and methods

### 2.1 Design

This qualitative descriptive study explored the cultural representations emerging from Italian and Spanish newspapers using the Emotional Text Mining (ETM) method (Greco, 2016a; Greco and Polli, 2020). ETM is an unsupervised text mining procedure grounded in a socio-constructivist approach and a psychodynamic model (Salvatore and Freda, 2011).

The emotional content in newspapers reflects shared emotional symbolizations related to events (Greco, 2016a,b), providing insights into human behavior that encompass both rational thinking and emotional functioning. Matte-Blanco (1975) identifies two parallel functions of the mind: the conscious mind, which categorizes reality, and the unconscious mind, which emotionally symbolizes these categories. These processes guide social adjustment and interactions (Fornari, 1976). Therefore, analyzing communication (text) serves as a reflection of behavior, social functioning, and shared emotional meanings, offering a pathway to understanding cultural representations of specific topics (Monaco et al., 2023).

Unlike traditional methods that define content *a priori* by applying predefined dictionaries (Bucci and Maskit, 2006), categories, or codes (Nandwani and Verma, 2021), ETM operates from a bottom-up perspective. It allows the content to emerge organically from the text itself, free from the constraints of predetermined categories. This inductive approach provides a more nuanced and context-sensitive analysis, capturing underlying emotional and symbolic representations that may not be immediately apparent through more rigid, top-down frameworks.

### 2.2 Data collection

Data were collected through a selection of newspaper articles from major newspapers in each country. The final corpus consisted of 1,735 articles. For Italy (Statista, 2021), we selected the newspapers “La Repubblica” and “Il Corriere della Sera,” from which we obtained a total of 1,287 articles. For Spain (Oficina de Justificación de la Difusión, 2021), we referred to the newspapers “El Pais” and “El Mundo,” from which we obtained 448 articles. These newspapers were chosen as they are the most widely read in their respective countries (Statista, 2021; Oficina de Justificación de la Difusión, 2021) and provided free access to their archives.

All articles published between January 1, 2013 and December 31, 2022, were considered for the collection, offering a 10-year time frame. This period was chosen because it was deemed appropriate for studying contemporary culture regarding the phenomenon in question. Only articles containing the translation of “assisted reproduction” (“fecondazione assistita” for Italian newspapers and “reproducción asistida” for Spanish newspapers) were included. The articles were downloaded from the respective newspapers’ online archives, and duplicate articles were excluded from the final group of articles.

A lexicometric evaluation of the indices of each corpus was then performed to assess linguistic richness (Italian corpus: Tokens = 754.932, Type/Tokens Ratio = 0.053, Hapax = 42.5%; Spanish corpus: Tokens = 573.801, Type/Tokens Ratio = 0.054, Hapax = 44.2%). It was noted that the hapax percentage should ideally be around 50%, and the type/token ratio should be below 0.2 for large corpora (Greco, 2016a).

### 2.3 Data analysis

T-LAB software (Cortini and Tria, 2014) is widely used in text analysis studies (Gennaro et al., 2019). It was used for the various phases of applying ETM to each corpus, following five main stages.

Through the application of Emotional Text Mining, we identified three levels of communication: factors, main themes (clusters), and sub-themes (Greco, 2016a,b; Zolnoori et al., 2019). This method allows for an in-depth analysis of large document sets through an automated, bottom-up natural language processing approach. It identifies both the semantic level (meanings—word co-occurrence) and the semiotic level (signs—the symbolic matrix) (Greco and Polli, 2020). ETM enables clustering and factorial analysis through the processing of articles combined into a single corpus and selecting relevant terms (Fronzetti Colladon et al., 2023).

The first step involved cleaning and pre-processing the data, which included normalization, selection of multiword expressions, vocabulary construction, and corpus segmentation (Lancia, 2012). This was followed by lemmatization, with lemmas identified both automatically and manually, excluding low-frequency terms and words from the prompt, as well as removing overly frequent words that are predictable for the topic under study (Bolasco and De Mauro, 2021). The corpus was then fragmented into context units (Giuliano and La Rocca, 2012). We identified links between words to determine their symbolic matrix within the text. Next, we interpreted the factorial spaces based on word polarization and clusters within the cultural meaning space. The final interpretation of the factorial axes, based on absolute contributions (Née et al., 2014), involved six judges: four health psychology experts, one cultural research expert, and one dynamic psychology expert. These judges interpreted the factorial space and labeled each factor according to word polarization (Greco, 2016a), ultimately leading to the analysis and discussion of labels for the final interpretation.

## 3 Results

According to Greco and Polli (2020), three validation measures were used to identify clusters: the Calinski–Harabasz index, the Davies–Bouldin index, and the intraclass correlation coefficient indices (ICC). The analysis of the corpora produced three clusters and two factors for the Italian corpus, and five clusters and four factors for the Spanish corpus.

### 3.1 Italian results

The analysis classified 92.9% of elementary context units. The intraclass correlation coefficient identified the optimal partition into three clusters, located in a factorial space of two factors (see Supplementary Figure 1).

The first factor (Table 1), “The Ambivalence of ART,” places assisted reproduction at the negative pole, *Therapy*, as a purely generative technique without the affective aspect of the term “care,” with predominately medicalized/scientific terms highlighted. At the other pole, *Reparative Ethics*, medically assisted reproduction is set in a more ethical context that seeks to limit the power of science.

**Table 1:** The composition of each Italian factor and the related polarities.

Factor I				Factor II			
The ambivalence of art				Imagining the family			
(Pole−) Therapy	CA	(Pole+) Restorative ethics	CA	(Pole−) Prohibition	CA	(Pole +) Desire	CA
Oocytes	−0.020	Homosexual	0.016	Court	−0.022	To think	0.014
(ovociti)		(omosessuale)		(corte)		(pensare)	
Specialist	−0.010	Adoption	0.012	Sentence	−0.017	To tell	0.013
(tecnico)		(adozione)		(sentenza)		(raccontare)	
Healthcare	−0.009	Civil	0.010	Courthouse	−0.013	To fell	0.010
(sanità)		(civile)		(tribunale)		(sentire)	
Donor	−0.009	Court	0.010	Judge	−0.012	House	0.01
(donatore)		(corte)		(giudice)		(casa)	
Patient	−0.009	Judge	0.008	Council	−0.011	To live	0.008
(paziente)		(giudice)		(consulta)		(vivere)	

The second factor (Table 1), “Imagining the Family,” involves fantasizing about the ART family. At the negative pole, *Prohibition*, medically assisted reproduction emerges with a strong legal and ethical dimension. The background appears persecutory, causing a sense of anxiety and punishment due to laws and limitations that may represent past social patterns transmitted with certain rigidity. Italian legislation indeed determines how ART can be considered lawful or unlawful based on socially accepted family contexts. At the positive pole, *Desire*, there is a more dreamlike and affective dimension of the family image, but nothing indicating a deeper emotional connection.

In Table 2, the four Italian Clusters are illustrated.

**Table 2:** Clusters emerged from the analysis (the original Italian keywords are shown in parentheses).

Cluster	UC	Label, description, and textual examples	Keyword
1	36.85%	“Parental Myth”	To think (pensare)
			Father (padre)
			To tell (raccontare)
		The desire to create a traditional family.	To become (diventare)
			History (storia)
			To speak (parlare)
		*Those mother’s children so badly wanted. I agree that a child can be brought up in the love of one mother, one father or even same-sex people*	To see (vedere)
			Large / Big (grande)
			To be born (nascere)
			To live (vivere)
2	27.11%	“Moral Judgment”	Court (corte)
			Sentence (sentenza)
			Adoption (adozione)
		Enacting laws to protect us from fear of social change.	Homosexual (omosessuale)
			Courthouse (tribunale)
			Civil (civile)
		*Stepchild adoption, what the constitutional court may decide today The Constitutional Court’s decision on whether or not to admit the unconstitutionality findings raised by the Bologna Children’s Court could close the debate on stepchild adoption*	Judge (giudice)
			Politicy (politica)
			Council (consulta)
			Parliament (parlamento)
3	36.04%	“Healthcare procedure”.	Oocyte (ovociti)
			Specialist (tecnico)
			Doctor (medico)
		Delivering service through the clinical practice.	Donor (donatore)
			Health (sanità)
			Pregnancy (gravidanza)
		*The number of cryopreserved embryos increased by 29.9%, as did the number of embryo freezing cycles, while the number of oocyte freezing cycles continued to fall. Finally, the age of women accessing Pma continues to rise (36.7 years for level II and III fresh techniques)*	Hospital (ospedale)
			Genetic (genetico)
			Possible (possibile)
			Procreation (procreazione)

Only the first 10 lemmas for each cluster were considered.

The first cluster (Table 2), “Parental Myth,” describes the purpose of Assisted Reproductive Technology (ART) as the creation of the traditional family. The verbs (to think, to tell, to become, to speak, and to see) emphasize the process of constructing this ideal family. The desire is for the future child to be the one to whom family traditions are passed on. In this cluster, the image of the family through ART excludes the healthcare component, as only the emotional and ethical background emerges to reinforce the utopian aspect.

The second cluster (Table 2), “Moral Judgment,” represents the moral dimension of society surrounding ART. There is an apparent urgency to enact laws to define boundaries on the applicability of ART in other family contexts, which would involve social reorganization (adoption, homosexuality). The rigid, preordained social patterns are transmitted and represented through institutions (Court, Courthouse, and Council) and laws (sentence, judge), creating a sense of anxiety and punishment, as well as stagnation.

The third cluster (Table 2), “Healthcare Procedure,” outlines the purely technical and medical aspect of ART. No emotional aspect regarding care is mentioned; rather the figures or institutions involved in healthcare (specialist, doctor, and hospital) and the figures related to the biological materials required for reproduction (oocytes, donor, and genetic) are highlighted.

### 3.2 Spanish results

The analysis classified 99.2% of elementary contexts. The intraclass correlation coefficient identified the optimal partition into five clusters, which are organized in a factorial space consisting of four factors (see Supplementary Figure 2).

The first factor (Table 3), “Progress,” describes assisted reproduction on the negative pole as *Scientific*, focusing solely on the scientific elements involved in the study of ART. On the positive pole, *Community/Legislative* terms reflect legislative regulation that protects ART in the social and communal dimension. In this case, progress would ensure and define the rights of all those who wish to resort to assisted reproduction regardless of their sexual orientation.

**Table 3:** The composition of each Spanish factor and the related polarities.

Factor I				Factor II				Factor III				Factor IV			
Progress				Access to reproduction				Maternal age				Cost			
(Pole -) Scientific	CA	(Pole +) Community /Legislative	CA	(Pole -) Guarantee Healthcare	CA	(Pole +) Limits	CA	(Pole -) Age-related Infertility	CA	(Pole +) Diagnosis and Treatment	CA	(Pole -) Private clinical service	CA	(Pole +) Public Research	CA
Sperm	−0.014	Law	0.022	Healthcare	−0.053	Parents	0.014	*In vitro*	−0.016	Cell	0.022	Patients	−0.057	Healthcare	0.017
(Esperma)		(Ley)		(Sanidad)		(padres)		(*in vitro*)		(célula)		(pacientes)		(sanidad)	
Research	−0.012	Legislation	0.017	Public	−0.034	Law	0.013	Fecudantion	−0.14	Research	0.018	(IVI)	−0.034	Sperm	0.014
(estudio)		(derecho)		(público)		(ley)		(fecundación)		(estudio)		(IVI)		(esperma)	
Cell	−0.009	Healthcare	0.011	Ministry	−0.016	Surrogacy	0.011	Age	−0.08	Sperm	0.016	Doctor	−0.031	Cell	0.013
(cèlula)		(sanidad)		(ministerio)		(subrogada)		(edad)		(esperma)		(doctor)		(célula)	
Risk	−0.008	Surrogacy	0.011	Service	−0.014	Gestation	0.011	Get pregnancy	−0.008	Genetic	0.015	Fertility	−0.029	Public	0.013
(riesgo)		(subrogada)		(servicio)		(gestacíon)		(quedar)		(genética)		(fertilidad)		(público)	
Genetic	−0.007	Public	0.008	Hospital	−0.014	Relative	0.010	Maternity	−0.006	Law	0.012	Infertility	−0.029	Growth	0.007
(genética)		(público)		(hospital)		(familiar)		(maternidad)		(ley)		(infertilidad)		(desarrollo)	

The second factor (Table 3), “Access to Reproduction,” refers to how medically assisted reproduction in the Spanish context is depicted. On the negative pole, it is portrayed as *Guaranteed Healthcare*, with terms related to accessibility in both public and private settings of this reproductive technique, not only for citizens but also for those coming from outside. On the positive pole, limits are delineated regarding the possibility of surrogacy, which Spain has been contemplating for several years but has not yet legalized.

The third factor (Table 3), “Maternal Age,” is positioned on the negative pole as *Age-related Infertility*, with terms related to both natural maternity and the fact that women in recent years have been postponing the age at which they want to have a child. This pole defines maternity more as a physical problem for the couple, as it is understood as infertility. On the positive side, *Diagnosis and Treatment*, the terms that have emerged indicate the diagnosis of actual infertility in the couple or individual and the consideration of medically assisted reproduction as a possible solution for parenthood.

The fourth factor (Table 3), “Costs,” is divided into the negative pole as *Private Clinical Service,* with terms revolving around the word “patients.” It outlines a private sector that provides ART services, involving various professionals in the technique.

On the other pole, *Public Research*, the terms that appear indicate the dimension of research on ART conducted by public health. Therefore, this factor outlines ART purely from an economic perspective.

The first cluster (Table 4) titled “Birth,” is characterized by terms such as parents, maternity, baby, day, life, to reach, and month. These terms in the analysis indicate the anticipation and existence of a future child. This cluster is positioned between the dimension of scientific progress, which includes clinical studies on ART, the economic dimension of public research, and simultaneously, the legal and physiological limitations of access to reproduction.

**Table 4:** Clusters emerged from the analysis (the original Spanish keywords are shown in parentheses).

Cluster	UC	Label, description, and textual examples	Keyword
1	26.87%	“Birth”	Insemination (fecundación)
			Parents (padres)
			Maternity (maternidad)
		ART just as a possible means of giving life.	Baby (bebé)
			Day (día)
			*In vitro* (*in vitro*)
		*The first ‘test-tube baby’ born in a Madrid hospital is 30 years old today. La Paz was the first public center in Spain to set up an ‘in vitro’ fertilization program*	Life (vida)
			To reach (llegar)
			Mounth (mes)
			To decide (decidir)
2	20.69%	“Successful Healthcare Model”	Health service (sanidad)
			Public (público)
			Ministry (ministerio)
		Spain’s vision of its healthcare model as an excellence.	Institute (centro)
			Health (salud)
			Hospital (hospital)
		*Health will finance assisted reproduction for ‘trans people with gestational capacity’. The minister signs an order to extend the service to lesbian, bisexual and single women […] The Ministry of Health has approved this Friday an order for the National Health System to pay for assisted gestation for all women*	Service (servicio)
			Euro (euros)
			Community (comunidad)
			Private (privado)
3	14.75%	“Assured Fertility”	Patientes (pacientes)
			Fertility (fertilidad)
			Medical (médico)
		ART seen as a product to be sold in private institutes.	Doctor (doctor)
			Problem (problema)
			Institute (centro)
		*Now, a project of the Valencian Infertility Institute (IVI) has been awarded the 300,000 euro Grant for Fertility Innovation (GFI) by a pharmaceutical company to add another technology to that of the Embryoscope and further improve the search for the perfect embryo that obsesses specialists and their patients.*	To explain (explicar)
			Infertility (infertilidad)
			IVI (IVI)
			Hospital (hospital)
4	21.76%	“Missing Law”	Law (ley)
			Relatives (familiar)
			Gestation (gestación)
		The absence of the surrogacy law in Spain as a topic of discussion.	Legislation (derecho)
			Person (persona)
			Subrogate (subrogada)
		*Families with children born through surrogacy have denounced this Friday the ‘dramatic’ situation they are facing because they are unable to register their children in the Civil Registry, and have demanded that the Ministry of Justice amend the law.*	Sexual (sexual)
			Parents (padres)
			Except (menos)
			Maternity (maternidad)
5	15.92%	“Ethics of research”	Research (estudio)
			Sperm (esperma)
			Genetics (genética)
		The progress of genetic research on controlling risks but also its limits.	Diseas (enfermedad)
			Cell (célula)
			Growth (desarrollo)
			Risk (riesgo)
		*Research: Before starting the experiment, for which they had to obtain permission from the British Human Fertilization and Embryology Authority and a bioethics committee, the researchers studied embryonic development in human embryonic stem cells and also in mouse models for months.*	Scientific (científico)
			Human (humano)
			Semen (semen)

Only the first 10 lemmas for each cluster were considered.

The second cluster (Table 4), “Successful Healthcare Model,” is characterized by terms that outline a model of excellent healthcare. The healthcare service provided involves various institutions, from the public sector (ministry, hospital) that handles legislative aspects and research, to the private sector that enables the application of ART with extended access not only for Spanish citizens but also for individuals from other countries (community, social, and access). This results in an economic return (euros).

The third cluster (Table 4), “Assured Fertility,” presents a set of terms that indicate ART from a business perspective. When a couple decides to seek treatment at one of the specialized centers in Spain, it is as if they are entering into a contract where the success of implantation and development is highly likely to be ensured. The cluster emphasizes the highly specialized and competent team that takes care of the patient solely from the perspective of infertility, without considering other psychological and contextual factors of the patient. The focus is solely on the desire to have a child, making both public and private facilities available, even if the woman’s age is no longer within the fertile range.

The fourth cluster (Table 4), “The Missing Law,” focuses the ART discussion on surrogacy and therefore on the impossibility of extending the issue of generativity due to the absence of legislation in this regard.

The fifth cluster (Table 4), “Ethics of Research,” showcases various terms that highlight the aspect of research progress, particularly in genetics, which enables the prevention of risks or diseases through the genetic study of sperm and ovum. Ethics refers to the limit of genetic perfection within which a scientist can venture.

## 4 Discussion

Assisted reproductive treatment plays a crucial role in healthcare, enabling reproduction for many who otherwise face challenges. However, access to ART varies across countries due to specific limitations. As Correia and Broderick (2009) highlight, cultural views on family, homosexuality, and ART significantly shape policy decisions regarding access. This cultural influence also affects how individuals experience ART.

This study compared cultural representations of ART in Spain and Italy using newspaper articles. Through Emotional Text Mining (ETM), we identified two factors and three clusters in the Italian sample, and four factors and five clusters in the Spanish sample, revealing notable differences. One significant finding is the representation of birth. In Italy, birth is closely linked to the traditional family structure—a mother, father, and child—suggesting that ART is primarily seen as a means to create this ideal family, which aligns with existing literature on social representations of family in Italy (Pozzi et al., 2022). In contrast, in Spain, birth is more broadly associated with parenthood and the celebration of life. This perspective emphasizes giving life and welcoming a newborn, reflecting a more expansive understanding of family.

These differences indicate how each country legitimizes ART: Italy focuses on the traditional family model, while Spain prioritizes the broader concepts of life and parenthood. This analysis underscores the impact of cultural representations on reproductive practices and access to ART in different contexts.

Considering the legislative context in which the use of ART is situated, the difference between the Italian and Spanish representations of justice is stark. In the Italian results, the law and the legal context emerge as almost persecutory dimensions and moral judgments, with boundaries that regulate and limit the use of alternative reproduction methods to natural reproduction (cluster 2). A possible explanation of these findings could be traced in previous analyses about Italian culture (Cordella et al., 2018), where the authority dimension emerged as a need to control the altered relationship between humans and nature. In the Spanish results, on the other hand, the law is linked to an ethical and communal dimension, where the law, instead of limiting, allows for the acquisition of rights for different actors in the population, involving the LGBTQ+ community (pole + factor 1). This is extremely important when considering how legislation plays a fundamental role in guiding access to ART. In Italy, ART is available only to cohabiting or married couples, while in Spain, it is accessible to both same-sex couples and single individuals. These legal differences are mirrored in the cultural contexts that have emerged in each country. This polarization (control vs. rights) could be explained considering the moral, ethical, and religious concerns implemented toward same-sex and single parenthood for a long time (DeLair, 2000; Peterson, 2005). Probably, the only ways to relate with this morality closure is just to adhere (Italy) or go against it winning rights (Spain).

It is also interesting to note that in the Spanish results, the public healthcare system is strongly present, while it does not emerge in the Italian results. The Spanish healthcare system emerges as “successful” (Cluster 2): public healthcare is connected to services and the economic dimension. It is important to note that this representation of the public healthcare system is in contrast with literature, which highlights how effectiveness of ART is greater in private than in public services (Alon and Pinilla, 2021; Castilla et al., 2009), suggesting a process of idealization of this field. Related to this, scientific research emerged as a creator of development for the community (positive pole factor 4), and access to healthcare seems to be represented as “guaranteed” (negative pole factor 2). In the Italian results, public health does not emerge; instead, ART is portrayed as a healthcare procedure lacking the emotional component (cluster 3). Regarding this, it is interesting to note that in the Spanish analysis, the third cluster (Assured Fertility) remains quite small (UC 14.5%). This cluster expresses the dimension of private healthcare and refers to ART center offers to the user who is also a paying consumer. This is particularly interesting when we consider the influence that private ART centers have in influencing knowledge and thus representations about reproduction in Spain (Mohammadi et al., 2019). This makes us think of the difficulty of the Spanish cultural system in thinking itself as a private company that produces economy through ART. It is no coincidence that the cluster related to public healthcare (Cluster 2) is much larger (20.69), confirming a greater propensity of the Spanish cultural system in thinking itself in a social and public dimension rather than a private and corporate one. It’s easier for the Spanish culture to refer to ART as a health care service rather than as a consumer good, despite the economic weight linked to ART in Spain.

In general, there are fewer factors and clusters in Italy (two factors and three clusters) compared to Spain (four factors and five clusters), and the Italian results seem less complex and crystallized than the Spanish ones. This general finding expresses the closure of the dialogue on ART in Italy compared to Spain. Interestingly, the dialogue in Italy is more crystallized despite having more articles published over the same period (1,278 in Italy versus 448 in Spain). This could suggest a greater need to address the topic in the Italian context, possibly due to the challenges posed by the influence of Catholic culture. It is interesting to note that the Spanish results also include the theme of surrogacy (positive pole factor 1, factor 2, cluster 4), indicating that the discussion on reproductive techniques is active even concerning new problems and possibilities that are not yet legal in the country. Surrogacy is presented in connection with words like *parents*, *law*, and *life*, suggesting that it is seen as another possible way of reproduction that is currently absent but discussed.

## 5 Conclusion

Assisted reproductive treatment is a highly complex topic that intersects with science, medicine, law, morality, religion, and individual experiences. We aimed to compare the cultures of ART in Spain and Italy, contrasting one of Europe’s most liberal and developed ART services with one of the countries with more restrictive laws. It is worth noting that approximately 3,350 ART cycles were performed in Spain in 2017 for people resident in Italy (Ministerio de Sanidad, Consumo y Bienestar Social, 2017).

Exploring the cultural representations of ART in Spain and Italy through Emotional Text Mining has allowed us to understand profound cultural differences that influence individual and social dimensions. The findings of this study could shed light on the cultural differences regarding ART and its relationship with the legal field and service provision. It would indeed be desirable to publish the results in Italian journals to disseminate and highlight the cultural gap that emerges from the present studies. Dissemination of the results in the Italian context could foster a more in-depth discussion and recognition of the cultural dimensions at play. These results could be discussed with policymakers and taken into account for the drafting of guidelines by ethics committees regarding ART. This could help achieve more adequate regulation and a better quality of health services in the countries, consequently leading to a better quality of life for people seeking parenthood.

Italy, in particular, could benefit from this, as its culture surrounding ART appears limited and limiting.

Furthermore, opening up the debate could enable both the population to become more aware of the possibilities and limitations of reproduction, and society to address this issue.

## 6 Limitations

This study has some limitations that need to be considered for future research. Firstly, the study focuses on only two newspapers per country. The newspapers considered cover a center-left (*La Repubblica*) and centrist (*Il Corriere della Sera*) position for Italy, and a center-left (*El Pais*) and center-right (*El Mundo*) position for Spain; future studies could include a broader range of newspapers, ensuring a balance according to their political orientation to examine how this influences the representations conveyed. Additionally, a time series analysis could be conducted to track the trend of conveyed representations over time and determine if they are influenced by specific historical periods and contexts.

Another limitation is the use of a specific methodology to analyze the corpus. It would be valuable for future research to apply other text analysis methodologies, such as ACASM (Gennaro et al., 2019), and compare the results.

Moreover, using only newspapers as a proxy for the culture of the countries may reflect the culture of a portion of the population, while younger individuals may be better represented through social media platforms (Instagram, TikTok, etc.). Other strategies such as interviews and ethnographic observations could be useful in future research to explore how the population experiences and represents the topic of ART.

## Data Availability

The original contributions presented in the study are included in the article/Supplementary material, further inquiries can be directed to the corresponding author.
