# Supplementary figures and images for: Analyzing assisted reproductive treatment representations in Italy and Spain through newspapers
Source: Front Psychol. 2024 Oct 23;15:1451663. doi: 10.3389/fpsyg.2024.1451663 (PMC11537878; doi:10.3389/fpsyg.2024.1451663)

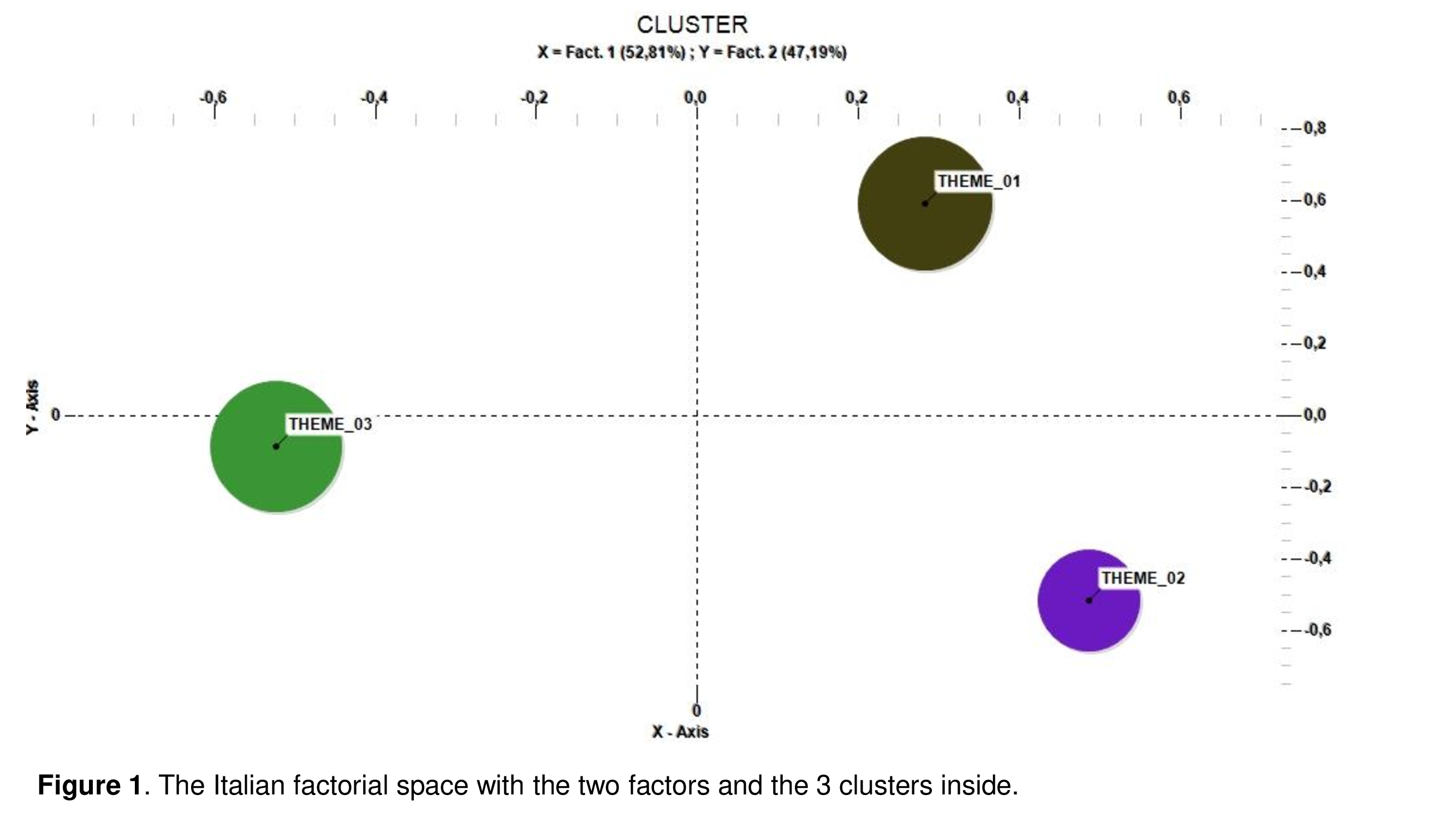

Supplement: Supplementary file 1 [file Image_1.JPEG]

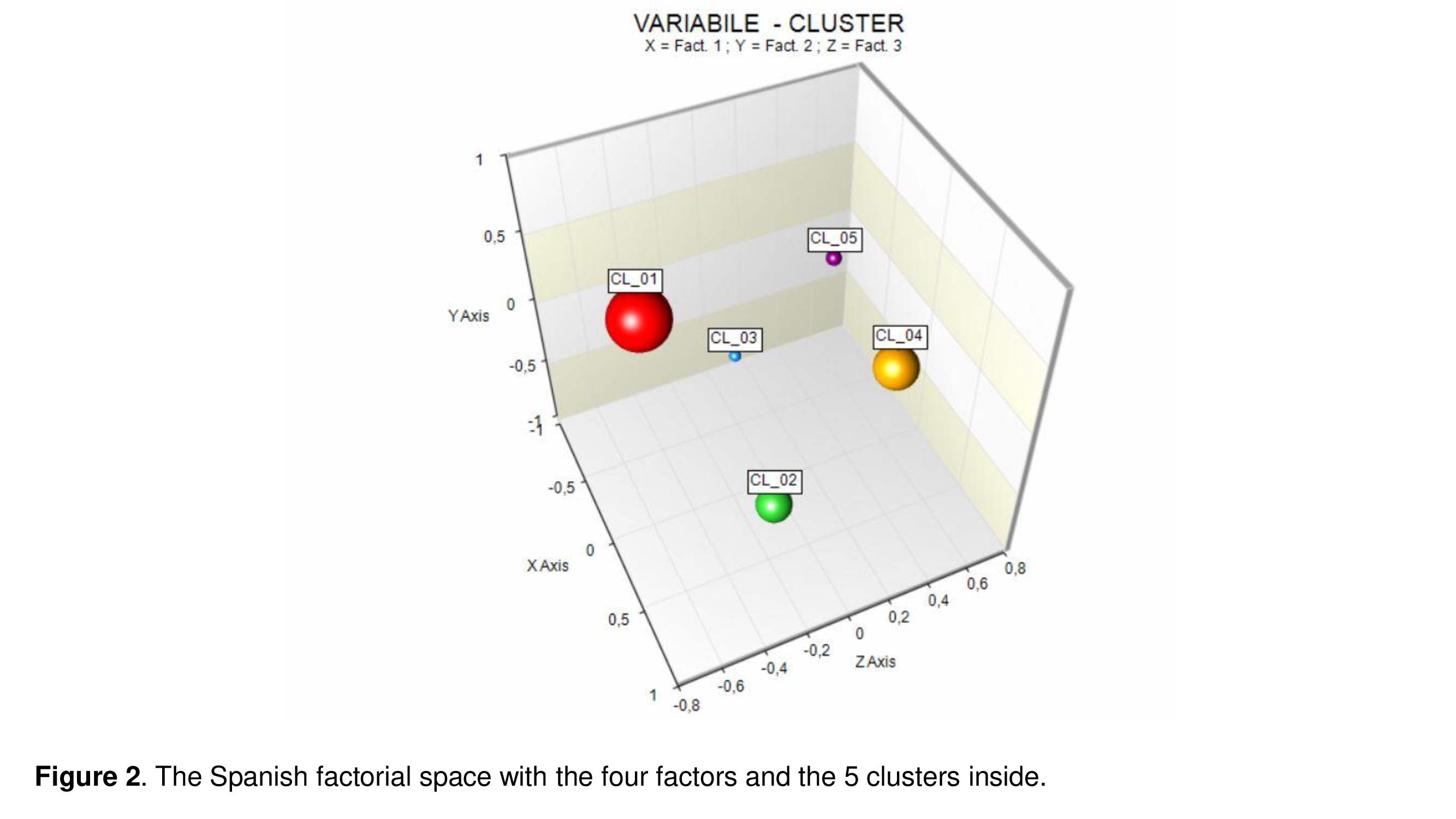

Supplement: Supplementary file 2 [file Image_2.JPEG]
